# Letter to the editor: Modified Stoppa as an alternative surgical approach for fixation of anterior fracture acetabulum: a randomized control clinical trial

**DOI:** 10.1186/s13018-021-02231-w

**Published:** 2021-01-29

**Authors:** Ahmed A. Khalifa

**Affiliations:** Orthopaedic and Traumatolgy Department, Qena Faculty of Medicine and University Hospital, South Valley University, Qena, Egypt

To the Editor:

Recently, I have read the research article entitled “Modified Stoppa as an alternative surgical approach for fixation of anterior fracture acetabulum: a randomized control clinical trial” [1] with great interest. Although I much appreciate the work done by the authors, however, major concerns emerged from just reading the title and the abstract.

The authors reported a “comparative study” where they compared a group of acetabular fractures operated through a modified Stoppa approach with a “historical” group of patients operated through an ilioinguinal approach. How come that the authors called their study “a randomized control clinical trial”? How did they manage to do randomization?

The authors included 18 patients in group A and 20 patients in group B. They mentioned that during the study period (from 2015 to 2019), 88 patients attended to the trauma unit; however, the sum of all the patients they mentioned (included and excluded) does not count to 88. They reported that they had five patients lost during follow-up; however, they did not mention if those patients were lost from the total attending patients or from either group. The follow-up period was presented as a mean only without reporting the standard deviation.

Table 3 has no footnotes to indicate the terms used within the table (AC, BC, TS, Mdn).

The conclusion reported in the abstract is considered exaggerated, as reporting that the “Stoppa approach is the most convenient approach” for anterior acetabular fractures is very difficult to conclude with such few numbers of patients included within the study. Many reports showed that the newer less invasive pararectus approach might give results comparable to the modified Stoppa approach [2–5]. The also mentioned that the modified Stoppa approach improves visualization in the “lateral compression injuries” (which is a type of pelvic ring fractures not acetabular fractures), which was not mentioned in the fracture types included in their study.

The authors did not explain their conclusion that the modified Stoppa approach is recommended in developing countries as an alternative to the ilioinguinal approach.

I would like the authors re-check their study design and the numbers mentioned in their study. Rushing into firm conclusions from such small sample sized studies can be unrealistic.

## Data Availability

All relevant data are included in the manuscript.
